# Disease or not, aging is easily treatable

**DOI:** 10.18632/aging.101647

**Published:** 2018-11-17

**Authors:** Mikhail V. Blagosklonny

**Affiliations:** 1 Cell Stress Biology, Roswell Park Cancer Institute, Buffalo, NY 14263, USA

**Keywords:** gerossuppresants, senolytics, longevity, lifespan

## Abstract

Is aging a disease? It does not matter because aging is already treated using a combination of several clinically-available drugs, including rapamycin. Whether aging is a disease depends on arbitrary definitions of both disease and aging. For treatment purposes, aging is a deadly disease (or more generally, pre-disease), despite being a normal continuation of normal organismal growth. It must and, importantly, can be successfully treated, thereby delaying classic age-related diseases such as cancer, cardiovascular and metabolic diseases, and neurodegeneration.

## Endless debate on aging and disease

For decades, one of the most debated questions in gerontology was whether aging is a disease or the norm. At present, excellent reasoning suggests aging should be defined as a disease [1–7]. I tend to define aging a disease, even though it is the norm. Vladimir Dilman referred to aging as “normal disease” [8,9].

As I emphasized in my publications, aging is not programmed. I have explicitly stated as such even in my article titled "Aging is not programmed: genetic pseudo-program a shadow of development growth" (PMID: 24240128). Aging is a normal continuation of the normal developmental program, so it is NOT a program but a purposeless, unintended quasi-program [10–16]. Yet, aging is also a deadly disease because it inevitably leads to death.

Indeed, aging is “the sum of all age-related diseases” and this “sum is the best biomarker of aging” [17]. Aging and its diseases are inseparable, as these diseases are manifestations of aging. Of course, any one age-related disease can occur at a young age due to genetic and environmental factors. What is important is that aging is sufficient to cause all age-related diseases, sooner or later, without dependence on genetic or environmental factors [18]: if Alzheimer’s disease or type 2 diabetes is not diagnosed during ones life time, it is only because cancer or a stroke terminates life before Alzheimer’s diseases or type 2 diabetes can be diagnosed (and vice versa).

## Aging is the sum of pre-diseases and diseases

Aging is an increase in the probability of death due to age-related diseases, which are late manifestations of aging [18]. Diseases are preceded by pre-diseases. For example, diabetes is diagnosed when fasting glucose levels are higher than 125 mg/dl, while levels of 100 to 125 mg/dl are considered pre-diabetes. Remarkably, diabetic complications such as nephropathy and retinopathy often develop before type 2 diabetes itself (see for references [19]). Although not formally a disease, pre-diabetes is currently treated to prevent diabetes [20–23]. Moreover, pre-diabetes is initiated by underlying processes that we will call pre-pre-diabetes, which arise while fasting glucose levels and glucose tolerance are still normal, though insulin levels are increased (hyperinsulinemia), indicating mild insulin resistance [24]. Hyperinsulinemia in healthy adults with normal glucose levels is predictive of type 2 diabetes over a 24-year follow-up [25,26]. Normal glucose levels (<100 mg/dl) associated with hyperinsulinemia is pre-pre-diabetes [27]. Hyperinsulinemia may in turn be driven by mTOR signaling [19], which suggests a state of pre-pre-pre-diabetes in which both glucose and insulin levels are normal. The condition that we can call pre-pre-diabetes is associated with future diabetes, cardiovascular disease and the all cause mortality rate [28]. Preventive treatment with metformin has been initiated during these very early disease stages in obese adolescents [29].

Another example is hypertension (a disease), which is defined arbitrarily as blood pressure (BP) above 140/90 mmHg. Pre-hypertension (or borderline hypertension) is defined as BP below 140/90 mmHg but higher than 120/80 mmHg. BP tends to increase with age, and those whose BP has not yet reached 140/90 (disease), or even 120/80 (pre-disease), may still have higher BP than they did when they were younger [30]. Mortality is associated with BP, even if it is lower than 140/90 [31]. Both pre-hypertension and pre-diabetes are age-related pre-diseases. Likewise, the asymptomatic stages of Alzheimer’s disease are also pre-disease.

**Figure 1:**
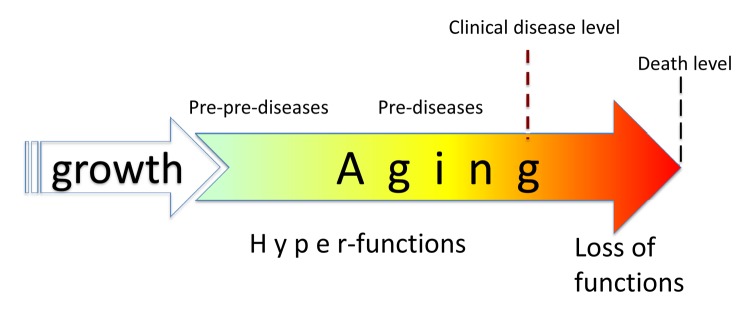
**Relationship between aging and diseases.** When growth is completed, growth-promoting pathways increase cellular and systemic functions and thus drive aging. This is a pre-pre-disease stage, slowly progressing to a pre-disease stage. Eventually, alterations reach clinical disease definition, associated with organ damage, loss of functions (functional decline), rapid deterioration and death.

In pre-diseases, abnormalities have not reached the arbitrary diagnostic criteria of the diseases. So, aging consists of progression from (pre)-pre-diseases (early aging) to diseases (late aging associated with functional decline). Aging is NOT a risk factor for these diseases, as aging consists of these diseases: aging and diseases are inseparable (Figure 1).

An aged appearance (e.g., grey hair, wrinkles, cushingoid body types and loss of muscles) are manifestations of pre-diseases. For example, an aged appearance may reflect hypercortisolism, sarcopenia, osteoporosis, skin pre-diseases and so on. And age-related skin lesions may herald pre-cancerous skin conditions [32].

## What is “healthy” aging?

What then is aging without diseases, so called “healthy” aging. “Healthy” aging has been called subclinical aging [33], slow aging [18,34] or decelerated aging [35], during which diseases are at the pre-disease or even pre-pre-disease stage. Diseases will spring up eventually. “Healthy” aging is a pre-disease state in which asymptomatic abnormalities have not yet reached the artificial definitions of diseases such as hypertension or diabetes. Instead of healthy aging, we could use the terms pre-disease aging or decelerated aging. Furthermore, decelerated aging can be achieved pharmacologically. For example, rapamycin decelerates aging, thereby making one healthier [36,37].

Currently, the term healthspan lacks clarity and precision especially in animals [38]. Although the duration of healthspan depends on arbitrary criteria and subjective self-rating, it is a useful abstraction. In theory, a treatment that slows aging increases both healthspan (subclinical period) and lifespan, whereas a treatment that increases lifespan (e.g., coronary bypass, defibrillation) is not necessarily increase healthspan (Figure 1 in reference [33]). The goal of both anti-aging therapies and preventive medicine is to extend healthspan (by preventing diseases), thus extending total lifespan.

## Preventive medicine: a step towards anti-aging medicine

Aging is the sum of diseases and pre-diseases. Treatments are generally more effective at pre-disease stages, associated with hyper-function, than at disease stages, associated with functional decline. As discussed in 2006, “rapamycin will prevent diseases rather than cure complications of diseases. For example, rapamycin will not repair broken bones but might prevent osteoporosis.” [10]. In fact, rapamycin prevents osteoporosis [39].

The goal of preventive medicine is to prevent diseases by treating pre-diseases. Thus, preventive medicine is a form of anti-aging therapy. Both preventive medicine and anti-aging therapy should prevent pre-diseases by treating “healthy” individuals. Some of the drugs used in preventive medicine include statins, aspirin, ACE inhibitors (e.g., lisinopril) and metformin, which can be repurposed as anti-aging drugs [40,41]. And *vice versa*, rapamycin, an anti-aging drug, may become a cornerstone of preventive medicine. As David Gems put it, “anti-aging treatment is any preventative approach to reduce late-life pathology. Its adoption would facilitate translation, since it would shift the emphasis to medical practice, particularly the introduction of preventative approaches.” [42].

## To treat what is treatable

The fact that aging is an obligatory part of the life of all organisms is not important. What is important is that aging is deadly and, most importantly, treatable. Consider an analogy. Is facial hair (beard) in males a disease? No of course, not. Still most men shave it, effectively “treating” this non-disease, simply because it is easily treatable. Is presbyopia (blurred near vision) a disease? It occurs in everyone by the age of 50 and is a continuation of developmental trends in the eye. It is treated as a disease because it is easily treatable with eye glasses. Unlike presbyopia, menopause in females is not usually treated because it is not easy to treat. Thus, the decision to treat or not to treat is often determined by whether it is possible to treat. It does not matter whether or not the target of treatment is called a disease.

## Aging is treatable

As the simplest example, calorie restriction (CR) slows aging in diverse organisms, including primates [43–50]. Similarly, intermittent fasting (IF) and ketogenic diet (severe carbohydrate restriction) extend life span in mammals [48,51–54]. CR (as well as carbohydrate restriction and IF fasting) improves health in humans [45,48,53,55–62]. However, CR is unpleasant to most humans and its life-extending capacity is limited. Nutrients activate the mTOR (molecular Target of Rapamycin) nutrient-sensing pathway [63–65] and, as we will discuss mTOR drives aging, inhabitable by rapamycin. Rapamycin-based anti-aging therapies have been recently implemented by Dr. Alan Green. https://rapamycintherapy.com

## Rapamycin and other rapalogs

Rapamycin (Rapamune/Sirolimus), an allosteric inhibitor of mTOR complex 1 [63,66], is a natural rapalog as well as the most potent and best studied rapalog. Rapamycin-analogs such as everolimus, temsirolimus (a rapamycin prodrug) and deforolimus/Ridaforolimus are also now widely used.

Rapamycin, everolimus and deforolimus slow geroconversion [67–75]. It has been predicted that rapamycin would slow aging in mammals [10,76]. Starting in 2009, numerous studies have demonstrated that rapamycin prolongs life in mice [75,77–99], even when started late in life [77,78,97–99], or administrated transiently or intermittently [77,88,89,95].

In these studies, rapamycin was most effective at high doses [88,89,93–96,100–103]. Its effect and that of everolimus lingers after their discontinuation [104], even after a single dose [105]. What appears to be important is to reach high peak levels using a single high dose [93,94].

In non-human primates, chronic and/or intermittent rapamycin improves metabolic functioning [106]. In a randomized controlled trial, middle-aged companion dogs administrated rapamycin exhibited no further side effects as compared to dogs receiving the placebo [107].

Millions of patients with various diseases and conditions (e.g., organ transplant recipients) have been treated with rapamycin (Sirolimus). Typical dose of rapamycin in organ-transplant patients is 2 mg/day. Rapamycin in a single dose of 15 mg was administrated to healthy volunteers without adverse effects [108]. Similarly, a dose of 8 mg/m2 (around 16 mg) was also well tolerated in healthy male volunteers [109]. What is amazing is that the placebo group reported more “side effects” such as astenia than did the rapamycin group [109]. In yet another study, comparison to placebo revealed no real everolimus-induced side effects in the elderly [104]. Moreover, everolimus improves immunity [110] and reduces infections in elderly healthy humans [104]. In placebo-controlled studies, side effects of rapamycin and everolimus are manageable with dose reduction and interruption. Discontinuation due to toxicity was uncommon [111]. In volunteers (aged 70-95 years, mean age of 80 years), treatment with 1mg/daily of rapamycin for 8 weeks was safe [112]. Matt Kaeberlein suggests that conventional doses of rapamycin maybe sub-optimal for maximum life-extension [113]. I agree with this opinion.

## Conventional drugs as anti-aging drugs

Metformin is used not only to treat diabetes but also pre-diabetes in order to prevent diabetes [20–23]. Metformin decreases insulin-resistance and body weight and prevents diabetes, cancer and cardiovascular disease [21,22,114–119]. It is expected that metformin would extend life and, in fact, metformin does decrease all-cause mortality [119,120]. Physicians generally do not think of metformin as an anti-aging drug, simply because it is expected that life will be extended, if diseases are prevented. In mice, metformin extends healthspan and lifespan [117,121–123]. It also extends the lifespan of *C. elegans* [124–127], which do not suffer from human diseases. Gerontologists think of metformin as an anti-aging drug [121–130], and metformin can be combined with rapamycin [131].

## Angiotensin II inhibitors

Angiotensin-converting enzyme (ACE) inhibitors (e.g., Captopril, Lisinopril, Enalapril, Ramipril) and Angiotensin II receptor blockers (ARB) (e.g., Valsartan, Telmisartan, Losartan) are widely used to treat hypertension, which is a typical hyperfunctional disease. Vasoconstriction, cardiomyocyte hypertrophy, beta- and alpha- adrenergic hyperstimulation all lead to high blood pressure (systemic hyperfunction), which, in turn can contribute to stroke, myocardial infarction and renal failure. ACE inhibitors and ARBs decrease vasoconstriction and prevent cardiac hypertrophy. They are life-extending drugs because they treat deadly diseases.

Notably, ACE inhibitors increase the lifespan in rodents with normal blood pressure [132–134], thereby acting as anti-aging drugs.

## Combinations of conventional drugs

Combinations of aspirin, statins, beta-blockers and ACE inhibitors are given to aging individuals to prevent cardiovascular diseases [135]. On the other hand, these drugs extend life span in rodents and Drosophila [136].

Typical combinations (polypill) include an antiplatelet agent (aspirin), a statin and two blood pressure-lowering drugs such as lisinopril and a beta-blocker [137,138]. Such combinations are estimated to reduce the 5-year incidence of stroke by 50% [139]. Aspirin, statins, ACE inhibitors, beta-blockers and metformin prevent some types of cancer and pre-cancerous polyps [116–118,140–146].

## Treating aging by preventing diseases or preventing diseases by slowing aging

As discussed, “aging is the sum of all age-related diseases” and this “sum is the best biomarker of aging” [17]. One could say that drugs prevent diseases by slowing aging. Alternatively, it could be said that prevention of diseases slows aging, which is the sum of all diseases and pre-diseases. If a drug prevents diseases, it will extend lifespan (apparently slowing down aging). If a drug slows down aging it will prevent diseases and extend healthspan [17,147].

As suggested “narrow spectrum anti-aging treatments (e.g. the cardiovascular polypill) could establish a practice that eventually extends to broader spectrum anti-aging treatments (e.g. dietary restriction mimetics)”. [42].

## CONCLUSION

It is commonly argued that aging should be defined as a disease so as to accelerate development of anti-aging therapies. This attitude is self-defeating because it allows us to postpone development of anti-aging therapies until aging is pronounced a disease by regulatory bodies, which will not happen soon. Aging does not need to be defined as a disease to be treated. Anti-aging drugs such as rapamycin delay age-related diseases. If a drug does not delay progression of at least one age-related disease, it cannot possibly be considered as an anti-aging drug, because it will not extend life-span by definition (animals die from age-related diseases). It has been suggested [17], “in order to extend life span, an anti-aging drug must delay age-related diseases. … Once a drug is used for treatment of any one chronic disease, its effect against other diseases … may be evaluated in the same group of patients.” Aging can be treated as a pre-disease to prevent its progression to diseases. Rapamycin-based combinations include conventional life-extending drugs, which are used to treat and prevent age-related diseases. These combinations could be combined with modestly low-calorie/carbohydrates diet, physical exercise and stress avoidance [40,41]. And this approach is actually being used now to treat aging at Alan Green’s clinic in Little Neck, NY: http://roguehealthandfitness.com/rapamycin-anti-aging-medicine-an-interview-with-alan-s-green-m-d/?print=pdf and https://rapamycintherapy.com
